# Gynaecologists' Views and Acceptability of Active Surveillance for Women Diagnosed With Cervical Intraepithelial Neoplasia 2: A Qualitative Study

**DOI:** 10.1111/ajo.70165

**Published:** 2026-07-30

**Authors:** Dan Luo, Ashleigh R. Sharman, Deborah Bateson, Megan Smith, Kirsten I. Black, Katy Bell, Kirsten J. McCaffery, Rachael H. Dodd

**Affiliations:** ^1^ The Daffodil Centre The University of Sydney, and Cancer Council NSW Sydney New South Wales Australia; ^2^ Susan Wakil School of Nursing and Midwifery, Faculty of Medicine and Health The University of Sydney Sydney New South Wales Australia; ^3^ Sydney School of Public Health, Faculty of Medicine and Health The University of Sydney Sydney New South Wales Australia; ^4^ Sydney Medical School, Faculty of Medicine and Health The University of Sydney Sydney New South Wales Australia; ^5^ Reproduction and Perinatal Centre, Faculty of Medicine and Health The University of Sydney Sydney New South Wales Australia; ^6^ Wiser Healthcare, Sydney School of Public Health The University of Sydney Sydney New South Wales Australia

**Keywords:** active surveillance, cervical cancer screening, cervical intraepithelial neoplasia/therapy, disease regression, qualitative research

## Abstract

**Background:**

Persistent oncogenic HPV infection can cause cervical epithelial changes leading to premalignant lesions such as CIN2. Active surveillance, a conservative option, reduces exposure to excisional risks while allowing timely treatment when needed. However, little is known about clinicians' perspectives, acceptability, and experience of offering active surveillance in the Australian healthcare context.

**Aims:**

To explore gynaecologists' perspectives, acceptability, and experiences in providing active surveillance to women diagnosed with CIN2.

**Materials and Methods:**

Individual semi‐structured interviews were conducted with obstetrics and gynaecology specialists and trainees in secondary and tertiary healthcare settings across Australia. Data were analysed using inductive thematic analysis.

**Results:**

Twenty‐five in‐depth interviews were conducted. Participants expressed positive views about active surveillance, regarding it as a safe option that does not increase long‐term cancer risk. When formulating management recommendations for CIN2, participants considered various interrelated diagnostic, patient, clinician, and policy‐level factors. To support shared decision‐making, participants used several communication strategies such as anatomical models and visual images to help address patient knowledge gaps and they provided patients with time and space to reflect on their preferred management plan. Participants identified strategies that could support offering active surveillance and patients' adherence to this, including enhanced clinician education and training, increased resourcing, and improved use of information technology to support follow‐up tests for active surveillance.

**Conclusions:**

A deeper understanding of multi‐level factors influencing clinicians' recommendations and implementation of active surveillance for CIN2 may inform the development of targeted interventions to address barriers and strengthen enablers, ultimately supporting wider offering of active surveillance.

## 1 Introduction

In Australia, the launch of the National Cervical Screening Program (NCSP) in 1991 led to a significant decrease in cervical cancer incidence and mortality [1]. A major update to the NCSP came into effect in December 2017, changing the target screening age from 20–69 to 25–74 years and switching from two‐yearly Papanicolaou (Pap) smears to five‐yearly Human Papillomavirus (HPV) testing, with reflex liquid‐based cytology (LBC) if oncogenic HPV types are detected [2].

Oncogenic HPV types, particularly HPV 16 and 18, cause over 70% of cervical cancer cases in Australia [3]. Persistent infection can induce cervical epithelial changes that progress to premalignant lesions [4]. These are classified as low‐grade (LSIL) and high‐grade (HSIL), with HSIL including cervical intraepithelial neoplasia (CIN) grades 2 (confirmed by p16 immunochemistry) and 3. In 2024, HSIL was diagnosed in 7.2 per 1000 women aged 25–74 undergoing cervical screening in Australia, with less than half being CIN2 [5].

Current Australian National Cervical Screening Program Guidelines (i.e., the guidelines) recommend treatment for CIN2 but note that for some cases of CIN2, a period of observation (active surveillance) may be acceptable [6]. As a conservative option, active surveillance allows time for HPV clearance while reducing exposure to the risks associated with excisional procedures and enabling timely treatment when necessary [7, 8]. This is supported by evidence showing high regression rates for biopsy‐confirmed CIN2 (ranging from 50% to 60%), and up to 65% in women under 25 years [9, 10, 11]. Active surveillance is offered in several countries, including United Kingdom, United States, and Nordic nations [11, 12, 13]. In Australia, the guidelines allow for 6–12 months of observation following histological diagnosis in selected cases, managed by experienced colposcopists [6]. If CIN2 persists or progresses to CIN3, active treatment is advised [6]. Despite this option, clinicians' perspectives, acceptability, and experience of active surveillance remain underexplored. This study therefore aims to address this gap.

## 2 Methods and Materials

### 2.1 Participants and Recruitment

Eligible participants were obstetrics and gynaecology (O&G) specialists and O&G trainees (i.e., registrars) practising in Australia. Purposive and snowball sampling were used to recruit a balanced mix of specialists and trainees across diverse geographic regions between September 2024 and February 2025. Participants were identified through the research team's networks, advertisements distributed by the Australia New Zealand Gynaecological Oncology Group, and posts on social media (e.g., X). Snowball sampling specifically targeted clinicians in suburban, rural, and remote areas to capture diverse perspectives.

Potential participants were contacted by email by several team members (DL, DB, KB, KM) and provided with a link to an Expression of Interest (EOI) form and Participant Information Statement. The EOI included a pre‐interview questionnaire on demographic and professional practice information. After completion, participants received a consent form, and interviews were scheduled at their convenience. This study was approved by The University of Sydney Human Research Ethics Committee (2024/HE000227).

### 2.2 Data Collection

Semi‐structured interviews were conducted between September 2024 and March 2025 using an interview guide (File S1) developed by the research team. Two researchers (DL, RD) conducted interviews via Zoom. Participants were informed of the study aims and encouraged to share their experiences of offering active surveillance to women with CIN2 during the interview. Interviews were audio‐recorded and lasted approximately 30 min on average. Data analysis occurred concurrently with data collection. When no new information emerged from the analysis of the 22nd interview, three additional interviews were conducted to confirm that data saturation had been reached [14].

### 2.3 Data Analysis

All interviews were transcribed verbatim and checked against audio recordings for accuracy. Inductive thematic analysis [15] was used to inform the analysis. Two researchers (DL, AS) independently coded 20% of transcripts using NVivo (Version 14, QSR International), with discrepancies resolved through discussion. One researcher (DL) then coded the remaining transcripts. Codes were organised into themes and reviewed with another researcher (AS), with potential themes and supporting extracts examined by the research team. Final themes were refined iteratively through team discussions.

## 3 Results

Twenty‐five gynaecologists participated in the study. Participant characteristics are presented in Table 1. Most participants demonstrated a strong understanding of the natural history of CIN2 by clearly describing information on the likelihood of regression. Participants' estimated proportion of their clinical caseload who were of childbearing‐age varied widely (10%–90%); with less variation in the estimated proportion who were diagnosed with CIN2 (2%–30%). All participants viewed active surveillance positively and considered it a safe management option for women with CIN2 that does not increase long‐term cancer risk. They expressed confidence in offering either active surveillance or immediate treatment (e.g., LLETZ) in certain circumstances based on the balance of relevant risk factors.

**TABLE 1:** Characteristics of study participants (*n* = 25).

Characteristics	*N* (%)
Age (years)^a^	43.2 ± 8.5
Gender	
Female	18 (72.0%)
Male	7 (28.0%)
Country of birth	
Australia	15 (60.0%)
Other	10 (40.0%)
Country where completed university medical education	
Australia	16 (64.0%)
Other	9 (36.0%)
State/territory of practice	
New South Wales	14 (56.0%)
Western Australia	7 (28.0%)
Queensland	2 (8.0%)
Northern Territory	1 (4.0%)
South Australia	1 (4.0%)
Location of practice	
Urban inner‐city	13 (52.0%)
Suburban	5 (20.0%)
Rural	5 (20.0%)
Urban inner‐city & Suburban	2 (8.0%)
Nature of practice	
Public	17 (68.0%)
Public & Private	5 (20.0%)
Private	3 (12.0%)
Practice setting	
Public hospital	14 (56.0%)
Private practice	3 (12.0%)
Academic, university‐based clinic	2 (8.0%)
Mixed practice settings	
Public hospital and private practice	4 (16.0%)
Private and university‐based clinic	1 (4.0%)
Public patients in private non‐for‐profit hospital	1 (4.0%)
Role of professional practice	
Obstetrics‐Gynaecology Specialist	8 (32.0%)
Obstetrician‐Gynaecologist Registrar	7 (28.0%)
General gynaecology specialist	5 (20.0%)
Gynaecologic‐Oncology specialist	5 (20.0%)
Main area of gynaecological expertise	
Obstetrics and Gynaecology	13 (52.0%)
Gynaecologic oncology	6 (24.0%)
General gynaecology	5 (20.0%)
Colposcopy, pelvic floor	1 (4.0%)
Years of experience treating women with cervical cancer or cervical abnormalities^a^	12.0 ± 8.7

^a^ Data is displayed as mean and standard deviation.

**FIGURE 1:**
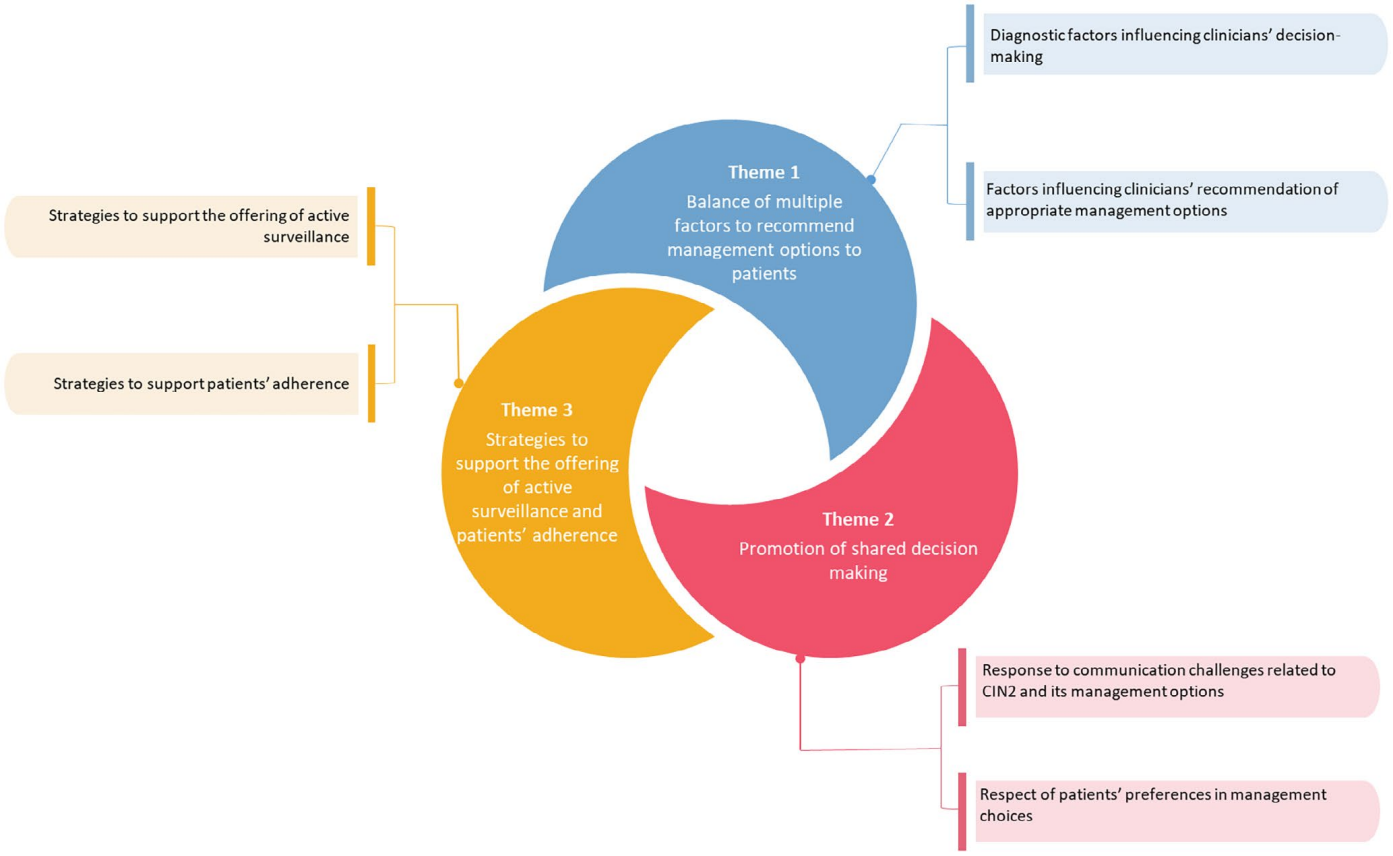
Themes and subthemes.

From our analysis, three themes, each with two subthemes, were identified (Figure 1). The illustrative quotations, including participant identifiers and professional roles, are presented in Table 2 to support the analysis of themes and subthemes.

**TABLE 2:** Themes, subthemes, and illustrative quotes.

Theme	Subtheme	Illustrative quotes
1. Balance of multiple factors to recommend management options to patients	1.1 Diagnostic factors influencing clinicians' decision‐making	*‘So probably biopsy is the most difficult, most important bit when it comes to diagnosing [CIN2].’ (Participant 7, trainee)*
		*‘…so if you have someone who's not an experienced colposcopist, then taking a biopsy in the right area might be difficult.’ (Participant 4, specialist)*
		*‘I think probably the real difficult one is that if you haven't got a good view of the transformation zone… it's a high grade on cytology, but I can't see anything [during the colposcopy].’ (Participant 18, specialist)* *‘…when you're doing colposcopy on someone, it is not the most comfortable procedure…you want to do a thorough exam, but it may be limited by patient discomfort.’ (Participant 4, specialist)*
		*‘…when you read the histopathology [report], you're not sure, what does he mean? Some say ‘Oh it's mainly CIN2’. so what is the rest? …you become a bit uncertain, is it CIN2 or more than this?’ (Participant 15, specialist)*
	1.2 Factors influencing clinicians' recommendation of appropriate management options	*‘…it [offering surveillance] depends on age…patients planning future fertility…not be smokers…have HPV other [not 16/18]… not have any evidence of immunosuppression… people are sometimes travelling for long time to get to the nearest town…to have colposcopy.’ (Participant 25, specialist)* *‘I mean they've been vaccinated, but there are still lots of strains of HPV you can get [which may lead to the CIN2].’ (Participant 10, trainee)*
		*‘I think a lot of them [clinicians]…are not up to date with the recent evidence [of active surveillance].’ (Participant 4, specialist)*
		*‘I think there is a real barrier if you don't believe that it can be safely managed in surveillance, then it will always be excision because that's what the clinician will recommend.’ (Participant 16, specialist)*
		*‘Sometimes you can get some tricky scenarios, where someone's come back as HPV other but possible high‐grade change [from the LBC test], then your biopsy actually doesn't show anything… with those ones…I do interact with [other] clinicians and ask them for their advice.’ (Participant 14, specialist)*
		*‘It's hard to know what to do…But generally, above the age of 30, I would tend not to encourage conservatism…you get uncomfortable [offering active surveillance] as you get further and further away from where a clinical trial drew its endpoints’ (Participant 2, specialist)*
		*‘…if there was a woman who was below 35 [and above 30], I probably would, with MDT and collaborative decision making, be happy to offer her active surveillance…’ (Participant 16, specialist)*
		*‘…to get a patient out of an Aboriginal community can cost $1000 or $1500, because they need the aircraft…need two‐ or three‐nights accommodation because the aircraft won't be going back for another two or three days…every year we go over budget. So they [state government] start looking at it closely to see where we can stop spending money.’ (Participant 11, specialist)*
2. Promotion of shared decision making	2.1 Response to communication challenges related to CIN2 and its management options	*‘…from a patient perspective…I think [there is] a challenge in understanding that, yes, you've told me there is cervical abnormal cells and to appreciate that it's actually not cancer and that it's actually safe to stay for six [months].’ (Participant 8, specialist)*
		*‘…we have a model in our room, like a uterus, cervix, vagina…I also draw on a piece of paper… where the [abnormal] cells are.’ (Participant 16, specialist)*
		*‘I explained to them that it's a pre‐cancerous [condition]…I'll say to them it's like a mole on your skin.’ (Participant 2, specialist)*
	2.2 Respect of patients' preferences in management choices	*‘…[for] patients planning future fertility…I would still offer surveillance.’ (Participant 25, specialist)*
		*‘I think some people would just be too worried about the fact they had CIN2 and they wouldn't consider conservative management because they [would] spend kind of six months being anxious.’ (Participant 23, specialist)*
3. Strategies to support the offering of active surveillance and patient adherence	3.1 Strategies to support the offering of active surveillance	*‘Probably more education…to the clinicians and the trainees…that it's an acceptable thing to do.’ (Participant 20, specialist)*
		*‘…if we have strong guidelines that support us in this group of women who've got ABCDE, you can safely offer surveillance for six months because we have detected that the actual progress into cancer rate is negligible.’ (Participant 9, trainee)*
	3.2 Strategies to support patients' adherence	*‘…if I needed them [patients] to come back at six months, I would actually get my receptionist to call and make an appointment. If they didn't answer, send them an email…[if no response again] send a text as well.’ (Participant 14, specialist)*

### 3.1 Theme 1 Balance of Multiple Factors to Recommend Management Options to Patients

Participants considered multiple factors, including diagnostic factors influencing clinicians' decisions on management plans (subtheme 1), and other factors shaping their decisions on recommended management options (subtheme 2) before making recommendations to patients.

#### 3.1.1 Diagnostic Factors Influencing Clinicians' Decision‐Making

Participants reported several key diagnostic factors influencing their decision making about CIN2 management, including quality of the biopsy sample and clarity of the histopathology report. Most participants noted that obtaining a sample from the area with the most severe lesion was the greatest challenge, as it depends on clinician's experience in identifying the correct area, the visibility of the transformation zone, and the need to balance patient discomfort with a thorough colposcopy examination.

Participants also reported that clarity of histopathology reports affects diagnostic certainty for CIN2. Some found the reports confusing, particularly when lacking a comprehensive overview of the biopsy sample, reflecting the challenges pathologists face in ‘distinguishing CIN2 and CIN3’ *(Participant 3, specialist)*.

#### 3.1.2 Factors Influencing Clinicians' Recommendation of Appropriate Management Options

Participants considered patient‐, clinician‐, and policy‐level factors when determining management options for patients. Key patient factors included age, fertility intentions, residential location, HPV type, immunosuppression and smoking status, prior cervical treatment, and ability to adhere to follow‐up tests. Participants were more likely to consider active surveillance for women of childbearing age with incomplete families, those with non 16 or 18 HPV types, absence of risk factors for cervical disease (e.g., immunosuppressed, not smoking), those with stable residence, and those living in urban areas. Most participants reported that HPV vaccination status did not influence their decisions, reasoning that the vaccine does not protect against all high‐risk HPV types and vaccinated individuals can still develop CIN2.

Participants' awareness and attitudes toward active surveillance, along with peer opinions, shaped their management recommendations. Although all participants were knowledgeable and willing to offer active surveillance, many peers still favoured immediate treatment due to a lack of awareness of the latest evidence or safety concerns. For complex cases (e.g., discordant histopathology and LBC results), most sought input from multidisciplinary (MDTs) teams or colleagues. Trainees typically developed management plans with senior staff (e.g., O&G specialists), whose guidance shaped their decisions.

Regarding policy factors, most participants acknowledged that guidelines were central to management decisions, while also recognising that evidence supporting active surveillance was largely derived from studies of younger women (< 30 years), in whom regression rates are relatively high. Participants' willingness to recommend active surveillance for those over 30 years of age varied and was often contingent on the multidisciplinary team's assessment of safety and appropriateness. They also considered the costs associated with offering active surveillance, particularly for patients in regional and remote areas, where limited state funding to support patients' access to specialist services in major cities constrained its feasibility.

### 3.2 Theme 2 Promotion of Shared Decision Making

Participants facilitated meaningful shared decision‐making with patients by actively addressing communication challenges related to CIN2 and its management options (Subtheme 1), and by respecting patients' preferences in management choices (Subtheme 2).

#### 3.2.1 Response to Communication Challenges Related to CIN2 and Its Management Options

During patient consultations, participants encountered several communication challenges, including patients' limited health literacy about active surveillance for CIN2, a lack of relevant educational materials, language barriers, and the need to manage negative emotions (e.g., fear, anxiety) following diagnosis.

To address these challenges, participants used various strategies and resources to improve patients' understanding of CIN2 and its management, and to reduce negative emotions. These included anatomical models, online images, and hand‐drawn illustrations to explain CIN stages, lesion location, and progression. Some also referred to Australian NCSP brochures and the guidelines to explain treatment. For non‐English‐speaking patients, participants used interpreter services, highlighting the need for multilingual materials. To ease concerns, participants emphasised that CIN2 is not cancer, using terms such as ‘pre‐cancerous condition’ or ‘abnormal cells’, and reassuring patients about possible regression.

#### 3.2.2 Respect of Patients' Preferences in Management Choices

In shared decision‐making, participants viewed their role as providing management options with associated risks and benefits while supporting patients to choose their preferred approach. They observed wide variation in patients' acceptance of active surveillance. Patients who had fertility desires, fear of treatment, or concerns about negative implications of treatment were more likely to accept active surveillance. Conversely, those anxious about CIN2, with a family history of cervical cancer, low tolerance for speculum examinations, concern about long wait times for colposcopy clinics, or who held certain cultural beliefs (e.g., linking HPV with ‘infidelity’ in Aboriginal communities) were inclined to favour treatment.

### 3.3 Theme 3 Strategies to Support the Offering of Active Surveillance and Patient Adherence

To facilitate wider implementation of active surveillance, participants suggested a range of strategies to support clinicians in offering active surveillance (subtheme 1) and conducting follow‐up tests (subtheme 2).

#### 3.3.1 Strategies to Support the Offering of Active Surveillance

Most participants suggested a need to enhance education and training on active surveillance for gynaecologists, including trainees, to raise awareness of its safety for managing CIN2. They also suggested that further clarification of objective factors (e.g., age, fertility intentions, residential location, immunosuppression, and smoking status) that could inform the safe provision of active surveillance in the guidelines would increase clinicians' confidence in recommending it.

#### 3.3.2 Strategies to Support Patients' Adherence

Participants consistently agreed that implementing active surveillance placed an additional burden on the public health system, particularly given the long wait time for accessing colposcopy services. Therefore, they called for the allocation of additional resources (e.g., more colposcopy clinics, equipment, and staffing) to support patients' adherence to the follow‐up tests. Several also noted that information technology (e.g., telephone, email, text messaging) could help reduce loss to follow‐up.

## 4 Discussion

This study explored gynaecologists' perspectives, acceptability, and experiences of offering active surveillance for patients with CIN2. Our findings identified interconnected factors influencing clinicians' confidence in the accuracy of the CIN2 diagnosis and their management recommendations. We also identified communication challenges related to CIN2 and its management, strategies to address these challenges, and approaches to support clinicians' offering of active surveillance and patient adherence to follow‐up tests.

Our findings suggest that patient characteristics, including age, HPV type, fertility wishes, smoking and immunosuppression status, adherence to follow‐up tests, and residential location, influence clinicians' management recommendations for women diagnosed with CIN2. This is consistent with previous studies that have found these factors to be associated with the rate of regression from CIN2 (e.g., age, HPV type) [16, 17], risk of progression to cervical cancer (e.g., HPV type, smoking and immunosuppression status) [13, 18, 19], adherence to follow‐up tests (e.g., residential location) [20, 21], and potential obstetric complications (e.g., prior cervical treatment history) [22]. More importantly, this finding also reflects clinicians' uptake of evidence on active surveillance in decision‐making, especially for those who have not completed childbearing and those recently treated for CIN2 [6].

For women of childbearing age and those aged over 30, our findings showed considerable variation in the management options recommended by clinicians. Given that increasing age is associated with lower CIN2 regression and a higher 20‐year cumulative risk of invasive cancer with active surveillance versus immediate treatment [11, 16], clinicians should exercise caution when recommending active surveillance for women aged over 30. Further research is needed to identify patient characteristics indicating suitability for observation over time in this group. Our findings also suggest that HPV vaccination status is not a significant factor influencing clinicians' decisions, reflecting clinicians' awareness that vaccines do not protect against all oncogenic types. Consequently, clinicians should prioritise HPV types over vaccination status when determining management options.

Participants' awareness and attitudes toward active surveillance also influence their likelihood of offering this management option. Prior studies show that many clinicians are not up to date with current research, limiting the translation of evidence into practice [23]. Therefore, improving clinicians' education and training on effective CIN2 management and awareness of the Guidelines allowing for flexibility in some circumstances, an approach also emphasised by participants, is essential to increasing their confidence in offering active surveillance as a safe management option.

A lack of patient education materials on CIN2 management, including the active surveillance approach, was identified as a key barrier to facilitating patients' understanding of management options during shared decision‐making. These materials can enhance patients' health literacy and informed decision‐making [24], and their development and evaluation are warranted. Participants also reported challenges in managing patients' negative emotions following a CIN2 diagnosis. Two qualitative studies found that it was common for patients with CIN2 to experience anxiety at diagnosis, and that women with anxiety and fear of cancer progression were more likely to choose surgical treatment over active surveillance [25, 26]. Addressing patients' negative emotions is therefore key to improving the acceptability of active surveillance. In line with previous research, our analysis also showed that emphasising that CIN2 is not cancer can help alleviate these emotions [25].

Participants also recommended expanding colposcopy clinics and leveraging information technology to support follow‐up tests in active surveillance. These align with evidence showing increased demand for colposcopy services due to changes in cervical screening criteria and confusion or reluctance relating to the changes [27, 28]. Expanding colposcopy capacity could help triage patients under active surveillance and reduce the additional burden of gynaecologists in the public health system. Additionally, our findings on using information technology to promote patient follow‐up align with previous research demonstrating that cell phone and text message reminders were effective methods for improving patient adherence in healthcare settings [29].

Several limitations should be considered. Firstly, the findings may not represent all clinicians' views and acceptability of active surveillance across Australia, as participants in our study generally held positive views of active surveillance and were willing to offer it to selected patients assessed at lower risk of progression to cervical cancer. Additionally, most participants practiced in urban and suburban areas, with only 20% practicing in rural areas. Further research is needed to explore rural practitioners' perspectives of active surveillance and identify additional barriers to its implementation.

In summary, this study identified a range of factors influencing clinicians' recommendations for management of CIN2. Understanding these factors may inform targeted interventions to address barriers, reinforce facilitators, and support informed, consistent implementation of active surveillance, where appropriate. For women aged over 30, clinicians should consider individual characteristics, such as HPV type and fertility wishes, when determining suitability for active surveillance.

## Funding

This work received Seed funding from Sydney Cancer Partners via a grant from the Cancer Institute NSW (Grant ID 2021/CBG0002).

## Conflicts of Interest

The authors declare no conflicts of interest.

## Supporting information


**File S1:** Interview guide.

## Data Availability

The data that support the findings of this study are available on request from the corresponding author. The data are not publicly available due to privacy or ethical restrictions.
