# Supplementary material for: Gynaecologists' Views and Acceptability of Active Surveillance for Women Diagnosed With Cervical Intraepithelial Neoplasia 2: A Qualitative Study
Source: Aust N Z J Obstet Gynaecol. 2026 Jul 30;66(4):e70165. doi: 10.1111/ajo.70165 (PMC13422237; doi:10.1111/ajo.70165)
Supplement: Supplementary file 1 — File S1: Interview guide. [file AJO-66-0-s001.docx]

**Supplementary File 1: Interview Guide**

1. Firstly could you please confirm your professional role and tell me a little about where you work?

**Current management of CIN2**

2. Could you please briefly describe for me what CIN2 is?

- What are the implications of a diagnosis of CIN2 for the woman?

3. From your perspective, what are the current management options for women with CIN2?

- How do you feel about those various treatments you described?

4. What do you see as the advantages and disadvantages of those treatments?

- Survival benefit?
- Quality of life?

5. What is your role in the decision-making process about which management is best for each patient?

- How does your role interact with those of other clinicians?

6. What kinds of factors would you say influence those decisions about which management is best for each patient?

- Clinical characteristics that may affect prognosis or differentiate high risk from low risk cases?
- Patient characteristics (e.g. age, comorbidities)?
- Patient preferences?

The guidelines recommend:

*REC10.4: HSIL (CIN2) and observation*

- *In some circumstances, it may be acceptable to offer a period of observation (generally 6–12 months) to women who have a histological diagnosis of HSIL (CIN2), and this would usually be supervised by an experienced colposcopist or at a tertiary centre. Observation may be considered for:*
  - - *women who have not completed childbearing*
    - *women with discordant histology and LBC prediction of pLSIL/LSIL*
    - *women with focal minor changes on colposcopy and HSIL (CIN2) on histology*
    - *women recently treated for HSIL (CIN2).*

7. In your current practice, do you offer active surveillance?

And just so we’re on the same page, the active surveillance approach means not performing immediate surgery and instead closely monitoring the patient using 6 monthly co-tests and colposcopies, with the possibility of subsequent surgery if there’s any sign of progression.

• If so: In what circumstances do you consider that option?

• Can you describe an example of a patient for whom you’d offer it and when you would not?

8. What does/would an active surveillance schedule look like?

- Give protocol used in other countries – Denmark. What do you see as the main advantages/disadvantages of active surveillance for CIN2?
- Any concerns about risk factors – who do you think should be offered active surveillance
  - Age – any age not comfortable doing AS in?
  - HPV status – HPV16 and CIN2 less likely to regress
  - Concerns about loss to follow up
  - Future children
  - Lesion size
  - Type of transformation zone

10. As active surveillance implies that the lesion and HPV have been left untreated, do you have any thoughts about that? (in relation to)

- Long term cancer risk in those following active surveillance
- Need for increased follow up?

11. Do you think active surveillance would be acceptable to women?

- - Present our experimental study findings showing 79% of women would choose active surveillance over surgery

**Communicating with patients about CIN2**

It’s acknowledged that a diagnosis of CIN2 can be misinterpreted as cancer, so I’d like to ask you a little bit about that.

12. Could you please tell me about your approach to explaining to patients what the diagnosis of CIN2 means, or trying to help them understand that?

- what labels use, abnormal cells, precancer

13. What is your approach to explaining to patients what the management options are for their CIN2?

- Do you use educational materials or decision aids to communicate any of this information?

14. What are the main challenges when discussing CIN2 and deciding about treatment?

- What do you think is challenging from the patient’s perspective?
- What do you find challenging from a clinician’s perspective?

15. Can you talk me through some of the difficulties in diagnosing CIN2 and how this would affect the offer of active surveillance?

**Childbearing age (25-45)**

We’re particularly interested in management of CIN2 for women of childbearing age. So I’d like to touch on some of my questions again, but this time asking you to specifically think about the patient population of women aged 25-45.

16. Of all the patients you see each year, roughly how many or roughly what proportion are aged 25-45?

• And what about among those with CIN2 specifically? (Among people aged 25-45, roughly how many or roughly what proportion are with CIN2)

17. For these women, how do you see the management options and their main advantages and disadvantages?

18. What are the key factors that influence treatment decisions for patients aged 25-45?

• Age, health/comorbidities, patient preferences?

- Would you want to offer those with HPV16 and CIN2 more aggressive treatment?

19. What are the main challenges when discussing CIN2 and deciding about treatment for this population?

20. How do you feel about active surveillance as a management option specifically for women aged 25-45?

- Any barriers to offering active surveillance
- Any facilitators

21. For women in the screening program who have been vaccinated, is there any differences in the management options provided to them?

22. Do you have anything further to add that we haven’t talked about today?

Thank you for your time.
